# Vitamin D and Child Neurodevelopment—A Post Hoc Analysis

**DOI:** 10.3390/nu15194250

**Published:** 2023-10-03

**Authors:** Megan D. Rodgers, Molly J. Mead, Caroline A. McWhorter, Myla D. Ebeling, Judy R. Shary, Danforth A. Newton, John E. Baatz, Mathew J. Gregoski, Bruce W. Hollis, Carol L. Wagner

**Affiliations:** 1 College of Medicine, Medical University of South Carolina, Charleston, SC 29425, USA; 2 Division of Neonatology, Department of Pediatrics, Shawn Jenkins Children’s Hospital, Medical University of South Carolina, Charleston, SC 29425, USA; 3 Darby Children’s Research Institute, Medical University of South Carolina, Charleston, SC 29425, USA; 4 Division of Biostatistics, Department of Public Health Sciences, Medical University of South Carolina, Charleston, SC 29425, USA

**Keywords:** cholecalciferol, neurodevelopmental assessments, longitudinal follow-up, pregnancy, offspring, maternal health outcomes

## Abstract

Introduction: Vitamin D (VitD) has been shown to impact neurodevelopment. Studies have shown that higher 25-hydroxy-vitamin D (25(OH)D) concentrations (the indicator of vitD status) may be associated with better neurodevelopmental outcomes, although current data are conflicting. This study examined the relationship between total circulating 25(OH)D concentrations and neurodevelopmental outcomes in 3–5-year-old (3–5 yo) children. Methods: In this study, pregnant women were randomized to receive 400 (standard dose), 2000, or 4000 IU vitD_3_/day. Offspring then underwent the Brigance Screen at 3–5 yo. The 25(OH)D concentration was measured at birth and 3–5 yo. Relationships between Brigance scores and 25(OH)D and Brigance scores and vitamin D binding protein (VDBP) genotype were examined. Results: Higher 25(OH)D at the time of testing was associated with better overall performance on neurodevelopmental testing as measured by the Brigance quotient (B = 0.208, *p* = 0.049). Scores were then broken down into sub-scores. Children born to mothers in the 2000 IU/day group scored higher on the Brigance language component of the assessment versus the standard dose group (B = 4.667, *p* = 0.044). The group of children who had the Gc1f-1s or Gc1f-2 genotypes scored higher on the Brigance academic component (B = 9.993, *p* < 0.001) and lower on the Brigance language component versus the 1f1f genotype (B = −9.313, *p* < 0.001). Children with the Gc1s-1s, Gc1s-2, or Gc2-2 genotypes also scored lower than the Gc1f-1f genotype (B = −6.757, *p* = 0.003). Conclusion: These results suggest that higher 25(OH)D concentrations early in life and higher doses of maternal vitamin D supplementation during pregnancy may have a positive association with neurodevelopmental outcomes. This study also suggests that the VDBP genotype is associated with neurodevelopment and differentially affects various fields of neurodevelopment.

## 1. Introduction

Vitamin D is well known as a modulator of calcium and bone homeostasis, but recent studies suggest that it may play a larger role as a neuroactive steroid affecting brain development and adult brain function [1]. In adults, vitamin D has been shown to play a role in several neurodevelopmental and neuropsychiatric conditions, and supplementation with vitamin D has been shown to improve symptoms of some of these diseases, including Alzheimer’s disease and attention deficit hyperactivity disorder [2,3].

In children, one condition having the most evidence for vitamin D’s relationship with neuropsychiatric disorders is autism spectrum disorders. A recent review details several studies that have found relationships between lower 25-hydroxy-vitamin D concentrations (25(OH)D) and an increased risk for the development of autism spectrum disorders in children and adolescents [4]. Maternal vitamin D during pregnancy has been linked to the development of autism spectrum disorder in offspring [5,6], while vitamin D supplementation during pregnancy in mothers with an existing child with autism spectrum disorder has been shown to result in reduced rates of recurrence in subsequent pregnancy [7]. A recent review summarized that there is a growing amount of evidence that vitamin D deficiency during pregnancy puts offspring at increased risk of developing neurological disorders, including autism spectrum disorder, behavioral disorders, schizophrenia, depression, and multiple sclerosis [8].

There is also a growing body of evidence that hypothesizes that vitamin D status during pregnancy may impact offspring brain development and morphology. Animal studies have demonstrated links between maternal vitamin D depletion and changes to offspring brain morphology and reduced gene expression of the forkhead box protein P2 (Foxp2) in the brain, which is critical for the development of speech and language in humans [9]. In a mouse model of immune inactivation, maternal vitamin D status was protective against the damaging effects of maternal immune activation on mesencephalic dopaminergic neurons, suggesting that vitamin D may play a neuroprotective role during fetal brain development [10]. A prospective study of children born to mothers with vitamin D deficiency during pregnancy found that the children studied had less cerebral gray and white matter volumes and smaller surface areas [11].

Several reports diverge from this hypothesis, however, including a systematic review that concluded that maternal vitamin D deficiency in the first or second trimester was associated with worsened socio-emotional development scores in offspring, while two others found that lower maternal 25(OH)D was associated with poorer language outcomes in children [12,13,14]. Additionally, another report examining the effect of vitamin D supplementation with 2800 IU/day during pregnancy on offspring neurodevelopment found that supplementation was not associated with differences in child neurodevelopment [15]. While one study found that higher cord blood vitamin D status was associated with better problem-solving abilities [16], other reports have contradicted this finding, showing little-to-no effect of vitamin D status or supplementation on neurodevelopmental outcomes in young children [17,18,19,20,21].

Another factor that is important to consider when examining the effects of vitamin D is the vitamin D binding protein (VDBP) genotype. The majority of vitamin D in the body circulates bound to VDBP, and recent studies have suggested that VDBP, also called group-specific component (GC), may play an important role in various pathologies, including several forms of cancer, thyroid diseases, obesity, diabetes, and several other pathologies [22]. VDBP is encoded by the GC gene on chromosome 4 and has three common alleles—Gc1f, which is the wild-type allele, GC1s, and GC-2, which can combine to form six different possible genotype combinations—Gc1f-1f, Gc1f-1s, Gc1s-1s, Gc1s-2, Gc1f-2, and Gc2-2 [23]. Among children who received the recommended daily allowance of vitamin D in their diet, those with the Gc1s allele had the highest concentrations of circulating 25(OH)D, while those with the Gc1f allele were more likely to be vitamin D insufficient [24]. One recently published paper showing associations between VDBP genotype, specifically the presence of the Gc1f allele, and worsening severity of autism spectrum disorder suggests a link between VDBP genotype and neurodevelopment [25]. However, the potential link between VDBP genotype and neurodevelopment is not fully understood.

Given the current discrepancy in the literature regarding the role of vitamin D in cognition and neurodevelopment and the potential impact of the VDBP genotype on 25(OH)D concentrations, vitamin D status, and various pathological states, we sought to determine if there is a link between vitamin D status and VDBP genotype on neurodevelopment. We hypothesized that (1) supplementation with higher doses of vitamin D during pregnancy and thus 25(OH)D concentrations (the marker of vitamin D status) in offspring would lead to better neurodevelopmental outcomes and (2) that children with the Gc1s allele would perform better on neurodevelopmental assessments than those with the Gc1f allele. In this post hoc analysis of offspring enrolled in a prospective follow-up study of maternal vitamin D supplementation during pregnancy, we examined whether maternal vitamin D supplementation during pregnancy and the subsequent 25(OH)D concentrations in offspring would impact scores on neurodevelopmental assessments performed at 3–5 years old. Therefore, our first endpoint was to determine whether there is a relationship between 25(OH)D concentration and child neurodevelopment, and the secondary endpoint was to determine whether VDBP genotype plays a role in this relationship.

## 2. Materials and Methods

### 2.1. Study Design and Participants

**Figure 1:**
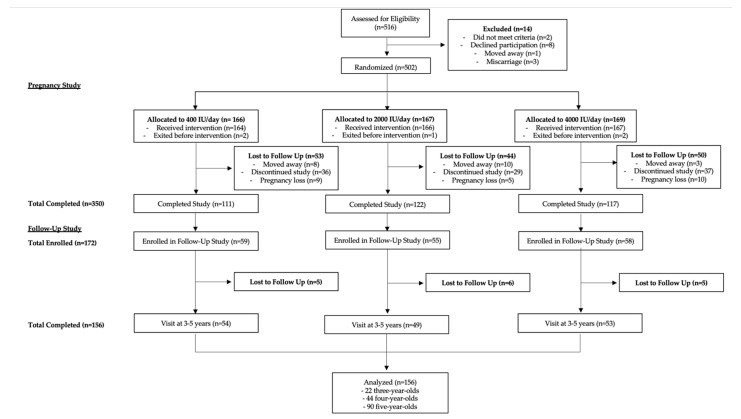
CONSORT Flow diagram outlining study procedures.

This study was conducted as a post hoc analysis of data from a follow-up study of a randomized clinical trial funded by the Thrasher Research Fund investigating the effects of vitamin D supplementation during pregnancy on child neurodevelopment. Both the pregnancy and follow-up studies were conducted at the Medical University of South Carolina in Charleston, SC. Both studies were conducted in accordance with the Declaration of Helsinki and approved by the institutional review board at the Medical University of South Carolina. The CONSORT flow diagram outlining study procedures is shown in Figure 1.

### 2.2. Pregnancy Study Design

The pregnancy study (HR#10727; clinicaltrials.gov #NCT00292591), which preceded the follow-up study, recruited participants between 2004 and 2009. Pregnant women with singleton pregnancy and good general health with no preexisting chronic diseases were recruited between 12 and 16 weeks of gestation. Women were randomized to receive 400, 2000, or 4000 IU vitamin D_3_/day, which was continued for the duration of the pregnancy until delivery as previously described [26,27]. Blood samples were collected at baseline (12–16 weeks gestation) and again monthly until delivery to measure total serum 25(OH)D concentration (D_2_ and D_3_ forms). A total of 502 pregnant women were initially enrolled in this study, and 350 continued until delivery. Written informed consent was obtained from the participants in this study.

### 2.3. Follow-Up Study Design

Participants in the follow-up study were the offspring of mothers who participated in the pregnancy study detailed above. Of the 350 women who completed the initial study, 172 consented to allow their offspring to participate in the follow-up study funded by the Thrasher Research Fund (HR#19461). Recruitment took place between 2009 and 2013. Inclusion criteria included offspring aged 3 years 0 months to 5 years 11 months (3–5 years old) born to mothers in the pregnancy study described above. These ages were chosen because of the validation of the Brigance developmental screening and its ease of use in the research setting. The follow-up age was not the same for all subjects because the offspring were born over several years, and the follow-up study was conducted over a 3-year period. Of the 172 initially enrolled in the follow-up study, 156 completed follow-up visits between 3 and 5 years old. Within this group, 22 children were 3 years old, 44 children were 4 years old, and 90 children were 5 years old.

Blood samples were obtained from all offspring at birth. After birth, offspring were seen once a year for 1 h long follow-up visits. During these visits, blood samples were obtained to evaluate the total circulating 25(OH)D concentration. Additionally, offspring underwent yearly developmental testing using the Brigance Screen II, described below. Written informed consent was obtained from the parents/guardians for this study.

### 2.4. Sociodemographic and Clinical Characteristic Variables

Maternal race was self-reported via questionnaire and was defined as African American, non-Hispanic Caucasian, or Hispanic. The extent of maternal education was dichotomized as (1) less than college or (2) some college or greater. Marital status was defined as married or unmarried, which included single, divorced, or widowed. Insurance status was defined as private, Medicaid, or none. The feeding status of the infants was dichotomized as (1) exclusively breastfed or (2) exclusively formula-fed or mixed formula-breastfed. Small for gestational age (SGA) was defined using Fenton growth curves [28].

### 2.5. Neurodevelopmental Assessments

At each yearly follow-up visit, children were evaluated by examiners who were blinded to the treatment group using the Brigance Screen II, a validated neurodevelopmental assessment tool (Curriculum Associates, LLC, North Billerica, MA, USA). Children whose first language was Spanish were examined by a bilingual English and Spanish-speaking tester whose first language was also Spanish.

The Brigance Screen measures three domains of development—language, motor, and academic. The language domain measures both receptive and expressive language; the motor domain measures both fine and gross motor skills; and the academic domain measures literacy and mathematics. The results of the Brigance Screen were reported as a pass/fail score, an overall Brigance Quotient based on performance on the screen as a whole, and as individual scores for each of the domains measured (language, motor, and academic scores). The Brigance raw score was reported on the following scale: 9–97 for 3-year-olds, 4–100 for 4-year-olds, and 13–96 for 5-year-olds. The raw score was then input into a score calculator to give the overall Brigance Quotient.

### 2.6. Total Circulating 25(OH)D Concentrations

Infant blood samples were collected at delivery and again at the 3-to-5-year-old visits. Serum samples were stored at −20 °C and were then assayed in duplicate using the Diasorin commercial radioimmunoassay (Diasorin, Stillwater, MN) as previously described to measure the total 25(OH)D concentration [26]. Vitamin D status was determined based on previously published cutoffs from the Endocrine Society using a level of 25(OH)D < 20 ng/mL marking deficiency; 21–29 ng/mL as insufficiency; and >30 ng/mL as sufficiency [29]. While there is some disagreement in the literature regarding which guidelines to use to determine sufficiency, we have chosen to use the Endocrine Society’s definition because this study was designed with those cutoffs in mind.

### 2.7. Vitamin D Binding Protein Genotypes

Vitamin D Binding Protein (VDBP) genotype analysis was performed on whole blood samples obtained from offspring at birth. Three VDBP alleles were analyzed: Gc1F (rs7041-T/rs4588-C), Gc1S (rs7041-G/rs4588-C), and Gc2 (rs7041-T/rs4588-A) [30]. Genotypes were determined by restriction-fragment length polymorphism analysis; methods and genotype distribution in this study population were previously described in detail [24]. For statistical purposes, the VDBP genotype was grouped into 3 separate groups based on their association with vitamin D status: (1) Gc1f-1f genotype, (2) Gc1f-1s or Gc1f-2 genotype, or (3) Gc1s-1s, Gc1s-2, or Gc2-2 genotype).

### 2.8. Statistical Analysis

A total of 156 children were included in the final statistical analysis. For each of these children, one visit between the ages of 3 and 5 years where neurodevelopmental testing was performed and 25(OH)D concentrations were measured was analyzed. Results were described using the mean and standard deviation for continuous variables and the median for integers and for situations where data were not normally distributed. Results were considered significant if *p* < 0.05. Chi-square analyses and Student’s *t*-test were used to assess population characteristics.

The primary outcome measures of this study were neurodevelopmental assessment scores on the Brigance Screen in association with the treatment group. The secondary endpoint, VDBP genotype and its relationship with neurodevelopmental scores, was also assessed. Linear regression was used to evaluate these outcome measures. Variables were first examined in univariate models and were controlled for in multivariate models if univariate analysis was significant. We then reduced the model to include only those variables that were significant in multivariate analysis into a final, fully reduced model. Treatment groups remained in all multivariate models, including the fully reduced model, regardless of significance due to study design. Parameters measured included offspring birth 25(OH)D concentration, offspring 25(OH)D concentration at the time of testing (3–5-year follow-up visit), maternal college education, maternal marital status, sex of the child, breastfeeding status, race, small for gestational age, season at the time of testing (3–5 years old), insurance, and VDBP genotype. Additionally, maternal 25(OH)D concentration both at baseline and prior to delivery was correlated with offspring 25(OH)D at birth.

## 3. Results

### 3.1. Baseline Sociodemographic and Clinical Characteristics

Baseline sociodemographic and clinical characteristics of the study population are shown in Table 1. There were no differences between groups for any variables, including maternal race, marital status, maternal education, insurance status, child’s sex, feeding status, child’s APGAR score, child’s gestational age at delivery, and birth weight. Mean infant serum 25(OH)D concentration at birth was highly correlated with maternal 25(OH)D concentration at the first prenatal visit (r = 0.35, *p* < 0.0001, Supplemental Figure S1) and maternal 25(OH)D concentration 1 month before delivery (r = 0.56, *p* < 0.0001, Supplemental Figure S2). At the follow-up visit between 3 and 5 years old, 19.2% of children were vitamin D deficient. The 25(OH)D concentrations at birth were similar across VDBP genotypes, but at 3–5 years, 25(OH)D was significantly higher in offspring with the 1s1s, 1s2, or 2,2 genotype versus the 1f1f genotype (*p* < 0.001) (Supplemental Table S1).

### 3.2. Clinical Characteristics and Brigance Scores

In final, fully reduced models, higher 25(OH)D concentrations at the time of testing (3–5 years old) were associated with higher Brigance quotient scores (B = 0.21, *p* = 0.049, Table 2). Maternal college education was also associated with a higher Brigance quotient (B = 7.29, *p* = 0.001, Table 2). Brigance quotients were lower among Hispanic patients (B = −10.19 *p* < 0.001, Table 2).

Both Hispanic (B = −13.14, *p* < 0.001, Table 3) and African American races (B = −8.48 *p* = 0.005, Table 3) were associated with lower Brigance academic scores. The Gc1f-1s or Gc1f2 genotype was associated with higher Brigance academic scores versus the Gc1f-1f genotype (B = 9.99 *p* < 0.001, Table 3).

Brigance language scores were higher among children whose mothers were randomized to the 2000 IU/day treatment group during pregnancy (B = 4.667, *p* = 0.044, Table 4). Children of college-educated mothers scored higher on the Brigance language section than children of mothers who were not college-educated (B = 6.352, *p* = 0.001, Table 4). Testing in the spring was associated with better Brigance language scores (B = 4.163, *p* = 0.028, Table 4). The Gc1f-1s or Gc1f-2 genotype for VDBP (B = −9.313, *p* < 0.001, Table 4) and the Gc1s-1s, Gc1s-2, or Gc2,2 genotypes (B = −6.757, *p* = 0.003, Table 4) were both associated with poorer performance on the Brigance language section versus children with the Gc1f-1f genotype.

Male children scored lower on the Brigance motor assessment than female children (B = −2.033, *p* = 0.021, Table 5). Breastfed children scored higher on the Brigance motor assessment than non-breastfed children (B = 2.406, *p* = 0.011, Table 5).

## 4. Discussion

The primary aim of this study was to examine the relationship between vitamin D status early in life and neurodevelopmental outcomes. The secondary outcome was to examine the relationship between VDBP genotype and neurodevelopmental outcomes. In this study, we demonstrated that even when controlling for several factors that may affect vitamin D status, both maternal vitamin D supplementation and child vitamin D status were associated with higher scores on neurodevelopmental testing.

Specifically, we demonstrate that higher child 25(OH)D at the time of testing was associated with higher Brigance quotient scores even when controlling for socioeconomic factors, including maternal education, marital status, race, and VDBP genotype. These results agree with a recently published study which found that vitamin D status was associated with better problem-solving abilities [16]. Overall, however, our results disagree with the majority of recently published studies in children, which have mostly found no differences in neurodevelopmental outcomes based on vitamin D status as measured by total circulating 25(OH)D concentrations [18,19,20]. Interestingly, the opposite has been shown in adults with higher 25(OH)D concentrations generally being associated with better cognitive function, lower concentrations being associated with poorer cognition and the development of dementia and Alzheimer’s Disease, and supplementation is associated with improved cognitive function and decreased beta-amyloid among Alzheimer’s patients [2,31]. This discrepancy in the literature, both among studies examining children and the discrepancy between the impact of vitamin D in children versus older adults, highlights the need for future studies examining the potential impact of early vitamin D status on neurodevelopment and across the lifespan.

This study also found evidence to support the important role that maternal vitamin D status during pregnancy may play in child neurodevelopment. We found that children born to mothers who received the 2000 IU/day dose of vitamin D during pregnancy scored higher on the Brigance language section than those born to mothers receiving the standard prenatal dose of 400 IU/day, even after controlling for several factors, which may also impact vitamin D status. Interestingly, this pattern did not extend to children born to mothers in the 4000 IU/day group.

In the design of the original study, the 4000 IU vitamin D_3_/day supplement was chosen because it was the dose likely to raise circulating 25(OH)D concentration into the sufficiency range without putting the subject at risk for hypercalciuria and hypercalcemia, which was supported by these data [26]. When the study was completed, post hoc analyses of this cohort and two others in South Carolina showed significant differences in pregnancy comorbidities and improved neonatal and infant outcomes with the 4000 IU group [27,32,33,34,35]. Further studies by other investigators have also shown these positive effects of higher dose vitamin D supplementation on complications of birth when initiated in the first trimester [36,37,38,39,40,41,42,43]. It is of special note that a large vitamin D trial assessing the supplementation of 4000 IU/day to pregnant women to reduce childhood asthma, the VDDART trial, in the initial publication, found marginal significance for this proposed protection [39]. Reanalyses of these data, however, accounting for baseline 25(OH)D concentrations as is necessary for the conduct of nutrient studies [44], which is a major confounding factor, yielded results showing vitamin D supplementation offered clear protection toward asthma and wheeze development [45]. In fact, this study concluded that a 4000 IU/day supplement was insufficient and needed to be increased. The benefit of vitamin D repletion during pregnancy, then, is multifactorial and involves immune aspects that may extend to later health and neurodevelopment.

In this study, it is unclear why the neurodevelopmental benefit was limited to the 2000 IU/day group since supplementation is only a surrogate for increasing circulating 25(OH)D concentration. Similar findings of benefit were noted when the association of 25(OH)D concentration and neurodevelopment was analyzed. Supplementing pregnant women with 4000 IU/day with further investigation of this neurodevelopment is necessary to resolve this question.

It is also important to note that in this post hoc analysis, 38% of the original cohort was seen in longitudinal follow-up at 3-to-5 years of age, and as such, the groups in this post hoc analysis were not complete. Analyzing neurodevelopmental outcomes on the basis of 25(OH)D concentration during pregnancy, at birth, and at the time of follow-up in the offspring as a continuous variable is a more accurate assessment of vitamin D’s influence that is not seen with treatment groups that are plagued by nonadherence to protocol [44,45].

This pattern also agrees with previously published animal data, which found that maternal vitamin D depletion was associated with morphological changes in the regions of the brain that in humans responsible for speech and language production [9]. The results from the present study, coupled with the findings from this previous study, suggest that there may be a similar phenomenon seen in humans, providing further support for the importance of vitamin D sufficiency throughout pregnancy achieved with higher dose vitamin D supplementation in lieu of the current standard 400 IU/day. Other aspects of higher vitamin D dosing up to 4000 IU/day during pregnancy related to maternal immunity and preeclampsia [42,46] have been shown; however, neurodevelopmental considerations and their interface with immune development in this context have yet to be fully explored.

The present study also found an association between VDBP genotype and performance on several measures of the neurodevelopmental screening tool, even when controlling for vitamin D status, supporting the idea that VDBP genotype has a role in neurodevelopment that is independent of its effect on vitamin D concentration. Previous research has shown that the Gc1f allele is associated with the lowest levels of vitamin D status, while the presence of the Gc1s allele is associated with the highest levels of vitamin D status [24]. In this study, participants with the Gc1f-1s or Gc1f-2 genotype scored similarly on the overall Brigance quotient but better on the academic section and worse on the language section when compared to the Gc1f-1f genotype. This shows that, independently from vitamin D concentration, those with at least one allele other than the Gc1f allele scored better on the academic portion of the test. It has been shown that the various phenotypes of VDBP have differing affinities for vitamin D [23,34], and we hypothesize that the effect seen in the present study could be explained by differing affinities between the genotypes. This finding suggests that available vitamin D may affect both language and academic development. Other studies have suggested that VDBP genotypes may play a role in neurodevelopmental disorders. For example, a recently published study shows associations between the VDBP genotype, specifically the presence of the Gc1f allele, and the worsening severity of autism spectrum disorder [25]. The present study is consistent with findings from this previous study and lends support to the idea that VDBP genotype may be implicated as a factor affecting various neurodevelopmental disorders.

Together, these results suggest that there is a relationship between vitamin D status and neurodevelopment in children and support the notion that a higher dose of vitamin D during pregnancy would be beneficial for child neurodevelopment, specifically within the language domain. Additionally, our results support the idea that the VDBP genotype may affect child neurodevelopment. Interestingly, our study shows that VDBP genotype affects each domain of neurodevelopment in different ways and independently from vitamin D status, with the Gc1f-1s or Gc1f-2 having both a positive effect on academic domain scores and a negative effect on language domain scores. Together, these results add to the growing body of literature surrounding vitamin D and neurodevelopment and speak to the importance of vitamin D on the developing brain.

### Strengths and Limitations

The strengths of this study include the testing of several areas of neurodevelopment, including motor, academic, and language domains, using a validated instrument. Our findings add to a growing body of literature on this topic, supporting some recent studies but contradicting others. Additionally, the inclusion of total circulating 25(OH)D concentration in regression models as an indicator of vitamin D status, in addition to using the treatment group, helps mitigate the issue of adherence to supplementation, which plagues many studies with the inherent variability of vitamin D status at baseline as is with all nutrient studies [44,45].

Other strengths of the study include the detailed information about the mother’s health during pregnancy and of the offspring collected during annual research visits, as well as the diverse study cohort that included Black American and Hispanic mothers and their offspring. Additionally, while it is possible that differences in vitamin D status during pregnancy and early childhood could exist due to outside sources of vitamin D including sunlight or diet, the inclusion of total circulating 25(OH)D in models as an indicator of vitamin D status to nullify these potential confounders is ultimately a strength of the study.

Potential limitations of this study include the smaller sample size, specifically for individual genotypes for VDBP analysis. Additionally, while we were able to control for maternal education in the regression models and showed that the impact of vitamin D on neurodevelopment persisted, we were unable to measure parental intelligence, which could be a significant confounding factor. We also did not assess environmental factors such as food insecurity, housing, crowding, and potential environmental exposures. In addition, other nutrients that could impact neurodevelopment were not measured and thus represent a potential limitation of this study.

## 5. Conclusions

In summary, we demonstrate that higher 25(OH)D early in childhood is associated with higher scores on neurodevelopmental testing. We also link higher dose maternal vitamin D supplementation and higher maternal 25(OH)D concentrations during pregnancy with better language development in offspring. Additionally, our results illustrate that the various VDBP genotypes may affect different domains of neurodevelopment in different ways, with children who have the Gc1f1s or Gc1f2 genotypes having higher academic scores and lower language scores. Together, these results indicate the importance of ensuring vitamin D sufficiency early in life, provide support for the use of higher doses of vitamin D supplementation during pregnancy, and confirm the need for further studies investigating the potential role of genetics in the relationship between vitamin D status and neurodevelopment.

## Figures and Tables

**Table 1:** Baseline characteristics of the study population. * = significant at *p* < 0.05.

	400 IU/day	2000 IU/day	4000 IU/day	*p*
**Race** n(%)				
African American	16 (29.6%)	16 (32.7%)	18 (34.0%)	0.59
Caucasian	17 (31.5%)	10 (20.4%)	17 (32.1%)	
Hispanic	21 (38.9%)	23 (46.9%)	18 (34.0%)	
**Maternal Marital Status** n(%)				
Single	30 (56.6%)	26 (53.1%)	27 (50.9%)	0.84
Married	23 (43.4%)	23 (46.9%)	26 (49.1%)	
**Maternal Education** n(%)				
Less Than High School	6 (12.2%)	11 (23.9%)	9 (17.7%)	0.69
High School	9 (18.4%)	7 (15.2%)	9 (17.7%)	
College	34 (69.4%)	28 (60.9%)	33 (64.7%)	
**Insurance Status** n(%)				
Private	22 (41.5%)	13 (26.5%)	21 (39.6%)	0.38
Medicaid	11 (20.8%)	15 (30.6%)	16 (30.2%)	
None	20 (37.7%)	21 (42.9%)	16 (30.2%)	
**Sex of Child** n(%)				
Female	27 (50.0%)	20 (40.8%)	28 (52.8%)	0.45
Male	27 (50.0%)	29 (59.2%)	25 (47.2%)	
**APGAR Scores** (median, IQR)				
1-min	8.0 (IQR 8.0–9.0)	8.0 (IQR 8.0–9.0)	8.0 (IQR 8.0–9.0)	0.59
5-min	9.0 (IQR 9.0–9.0)	9.0 (IQR 9.0–9.0)	9.0 (IQR 9.0–9.0)	0.69
**Birth Characteristics** (mean + SD, range)				
Gestational Age (weeks)	38.8 ± 2.4 (Range 28.3–41.3)	38.7 ± 2.1 (Range 27.2–41.3)	38.8 ± 2.0 (Range 27.0–41.0)	0.94
Birth weight (g)	3174.9 ± 693.8 (Range 935.0–4961.0)	3411.6 ± 635.9 (Range 1113.0–4701.0)	3195.5 ± 664.3 (Range 948.0–4621.0)	0.14
# SGA (n, %)	9 (17.0%)	2 (4.1%)	5 (9.62%)	0.10
**Infant Feeding Status** n(%)				
Breastfed	35 (67.3%)	25 (52.1%)	32 (64.0%)	0.42
Formula Fed	9 (17.3%)	10 (20.8%)	6 (12.0%)	
Mixed	8 (15.4%)	13 (27.1%)	12 (24.0%)	
**25(OH) Vitamin D** (mean + SD)				
Maternal initial prenatal visit 25(OH)D (ng/mL)	24.3 ± 13.0	22.0 ± 7.7	21.8 ± 8.7	0.57
Maternal 25(OH)D (ng/mL) 1 month before delivery	32.5 ± 14.4	40.2 ± 15.2	44.7 ± 14.4	<0.001
Offspring Birth 25(OH)D (ng/mL)	17.2 ± 9.0 (Range 3.6–40.0)	22.2 ± 9.3 (Range 5.5–45.3)	27.7 ± 9.5 (Range 6.8–47.8)	<0.001 *
Offspring 3–5 year 25(OH)D (ng/mL)	30.2 ± 8.6	28.1 ± 10.83	27.7 ± 12.6	0.20

**Table 2:** Regression models for Brigance quotient. * significant, *p* < 0.05.

*BRIGANCE QUOTIENT*			
Characteristic	Univariate Model *B ± SE*|*p*-Value	Multivariate Model *B ± SE*|*p*-Value	Fully Reduced Model *B ± SE*|*p*-Value
**Treatment Group**			
400 IU/day	*Reference*	*Reference*	*Reference*
2000 IU/day	0.35 ± 2.73|0.90	2.17 ± 2.53|0.39	2.40 ± 2.47|0.33
4000 IU/day	−0.46 ± 2.63|0.86	−0.25 ± 2.61|0.92	−0.46 ± 2.55|0.86
**25(OH)D**			
Birth 25(OH)D	0.16 ± 0.11|0.15	0.05 ± 0.12|0.71	0.04 ± 0.12|0.71
3–5 Year 25(OH)D	0.36 ± 0.01|0.30	0.18 ± 0.11|0.01	0.21 ± 0.11|0.05 *
**Maternal Education**			
No College Education	*Reference*	*Reference*	*Reference*
College Educated	11.01 ± 2.05|<0.001	7.46 ± 2.27|0.001	7.28 ± 2.22|0.001 *
**Marital Status**			
Unmarried	*Reference*	*Reference*	
Married	6.66 ± 2.13|0.002	1.44 ± 2.49|0.56	
**Sex of Child**			
Female	*Reference*		
Male	−2.00 ± 2.18|0.36		
**Breastfeeding Status**			
Formula-fed	*Reference*	*Reference*	
Breastfed	6.79 ± 2.18|0.002	−0.83 ± 2.35|0.725	
**Race**			
White	*Reference*	*Reference*	*Reference*
Hispanic	−15.77 ± 2.38|<0.001	−17.37 ± 6.65|0.010	−10.19 ± 3.0|<0.001 *
African American	−7.53 ± 2.44|0.002	−3.10 ± 3.89|0.43	−3.42 ± 3.23|0.29
**Small for Gestational Age**			
AGA or LGA	*Reference*		
SGA	−1.10 ± 3.52|0.75		
**Season**			
Summer, Fall, Winter	*Reference*	*Reference*	
Spring	5.87 ± 2.17|0.008	2.29 ± 2.02|0.26	
**Insurance Status**			
Self-Pay	*Reference*	*Reference*	
Private Insurance	12.83 ± 2.35|<0.001	−8.59 ± 6.22|0.170	
Medicaid	6.67 ± 2.53|0.009	−8.19 ± 5.88|0.17	
**Vitamin D Binding Protein Genotype**			
1f1f	*Reference*		
1f1s or 1f2	−0.51 ± 2.92|0.86		
1s1s, 1s2, or 2,2	−1.29 ± 2.63|0.63		

**Table 3:** Regression models for Brigance academic sub-score. * significant, *p* < 0.05.

*BRIGANCE ACADEMIC*			
Characteristic	Univariate Model *B ± SE*|*p*-Value	Multivariate Model *B ± SE*|*p*-Value	Fully Reduced Model *B ± SE*|*p*-Value
**Treatment Group**			
400 IU/day	*Reference*	*Reference*	*Reference*
2000 IU/day	−4.37 ± 2.65|0.10	−3.74 ± 2.37|0.117	−2.49 ± 2.36|0.29
4000 IU/day	−3.92 ± 2.56|0.13	−4.90 ± 2.45|0.047	−3.85 ± 2.25|0.09
**25(OH)D**			
Birth 25(OH)D	0.24 ± 0.10|0.02	0.16 ± 0.11|0.15	
3–5 Year 25(OH)D	0.40 ± 0.09|<0.001	0.15 ± 0.09|0.11	
**Maternal Education**			
No College Education	*Reference*	*Reference*	
College Educated	6.96 ± 2.12|0.001	3.83 ± 2.14|0.08	
**Marital Status**			
Unmarried	*Reference*	*Reference*	
Married	8.46 ± 2.01|<0.001	2.01 ± 2.23|0.37	
**Sex of Child**			
Female	*Reference*		
Male	−0.80 ± 2.15|0.71		
**Breastfeeding Status**			
Formula-fed	*Reference*		
Breastfed	1.84 ± 2.20|0.40		
**Race**			
White	*Reference*	*Reference*	*Reference*
Hispanic	−12.60 ± 2.42|<0.001	−13.06 ± 5.95|0.03	−13.14 ± 2.38|<0.001 *
African American	−11.96 ± 2.48|<0.001	−5.06 ± 3.20|0.12	−8.48 ± 2.97|0.005 *
**Small for Gestational Age**			
AGA or LGA	*Reference*		
SGA	−3.58 ± 3.43|0.30		
**Season**			
Summer, Fall, Winter	*Reference*	*Reference*	
Spring	3.60 ± 2.16|0.01	1.79 ± 1.93|0.35	
**Insurance Status**			
Self Pay	*Reference*	*Reference*	
Private Insurance	10.92 ± 2.20|<0.001	0.40 ± 6.02|0.95	
Medicaid	−3.06 ± 2.37|0.20	−6.86 ± 5.67|0.23	
**Vitamin D Binding Protein Genotype**			
1f1f	*Reference*	*Reference*	*Reference*
1f1s or 1f2	9.42 ± 2.73|<0.001	7.90 ± 2.74|0.005	9.99 ± 2.91|<0.001 *
1s1s, 1s2, or 2,2	6.81 ± 2.48|0.007	4.19 ± 2.53|0.10	4.86 ± 2.93|0.10

**Table 4:** Regression models for Brigance language sub-scores. * significant, *p* < 0.05.

*BRIGANCE LANGUAGE*			
Characteristic	Univariate Model *B ± SE*|*p*-Value	Multivariate Model *B ± SE*|*p*-Value	Fully Reduced Model *B ± SE*|*p*-Value
**Treatment Group**			
400 IU/day	*Reference*	*Reference*	*Reference*
2000 IU/day	4.29 ± 2.52|0.09	4.61 ± 2.32|0.05	4.67 ± 2.29|0.04 *
4000 IU/day	2.75 ± 2.43|0.26	2.96 ± 2.22|0.18	3.13 ± 2.21|0.16
**25(OH)D**			
Birth 25(OH)D	−0.03 ± 0.11|0.76		
3–5 Year 25(OH)D	0.07 ± 0.10|0.49		
**Maternal Education**			
No College Education	*Reference*	*Reference*	*Reference*
College Educated	6.89 ± 2.02|<0.001	4.88 ± 2.16|0.03	6.35 ± 1.89|0.001 *
**Marital Status**			
Unmarried	*Reference*		
Married	−0.93 ± 2.06|0.65		
**Sex of Child**			
Female	*Reference*		
Male	0.42 ± 2.04|0.84		
**Breastfeeding Status**			
Formula-fed	*Reference*		
Breastfed	3.09 ± 2.08|0.14		
**Race**			
White	*Reference*	*Reference*	
Hispanic	−6.89 ± 2.40|0.005	−11.79 ± 6.12|0.06	
African American	2.27 ± 2.46|0.36	−1.81 ± 3.29|0.58	
**Small for Gestational Age**			
AGA or LGA	*Reference*		
SGA	2.49 ± 3.25|0.44		
**Season**			
Summer, Fall, Winter	*Reference*	*Reference*	*Reference*
Spring	5.42 ± 2.02|0.008	4.01 ± 1.89|0.04	4.163 ± 1.879|0.028 *
**Insurance Status**			
Self Pay	*Reference*	*Reference*	
Private Insurance	5.73 ± 2.31|0.014	−8.76 ± 6.17|0.16	
Medicaid	9.17 ± 2.49|<0.001	−6.05 ± 5.79|0.30	
**Vitamin D Binding Protein Genotype**			
1f1f	*Reference*	*Reference*	*Reference*
1f1s or 1f2	−10.09 ± 2.57|<0.001	−7.79 ± 2.93|0.009	−9.31 ± 2.45|<0.001 *
1s1s, 1s2, or 2,2	−7.60 ± 2.32|0.001	−5.63 ± 2.89|0.05	−6.76 ± 2.20|0.003 *

**Table 5:** Regression models for Brigance Motor sub-score. * significant, *p* < 0.05.

*BRIGANCE MOTOR*			
Characteristic	Univariate Model *B ± SE*|*p*-Value	Multivariate Model *B ± SE*|*p*-Value	Fully Reduced Model *B ± SE*|*p*-Value
**Treatment Group**			
400 IU/day	*Reference*	*Reference*	*Reference*
2000 IU/day	−0.80 ± 1.14|0.49	−0.29 ± 1.11|0.79	−0.39 ± 1.08|0.72
4000 IU/day	−0.68 ± 1.10|0.54	−0.70 ± 1.05|0.51	−0.75 ± 1.04|0.47
**25(OH)D**			
Birth 25(OH)D	0.05 ± 0.05|0.34		
3–5 Year 25(OH)D	0.06 ± 0.04|0.14		
**Maternal Education**			
No College Education	*Reference*		
College Educated	2.47 ± 0.91|0.008		
**Marital Status**			
Unmarried	*Reference*		
Married	2.13 ± 0.90|0.02		
**Sex of Child**			
Female	*Reference*	*Reference*	*Reference*
Male	−1.91 ± 0.90|0.04	−1.95 ± 0.89|0.03	−2.03 ± 0.87|0.02 *
**Breastfeeding Status**			
Formula-fed	*Reference*	*Reference*	*Reference*
Breastfed	2.94 ± 0.91|0.001	1.82 ± 1.08|0.09	2.41 ± 0.93|0.01 *
**Race**			
White	*Reference*	*Reference*	
Hispanic	−2.82 ± 1.11|0.01	−0.17 ± 1.50|0.91	
African American	−2.44 ± 1.14|0.03	−0.44 ± 1.44|0.76	
**Small for Gestational Age**			
AGA or LGA	*Reference*		
SGA	−1.29 ± 1.46|0.38		
**Season**			
Summer, Fall, Winter	*Reference*		
Spring	0.31 ± 0.93|0.74		
**Insurance Status**			
Self Pay	*Reference*		
Private Insurance	1.67 ± 1.07|0.12		
Medicaid	0.53 ± 1.15|0.65		
**Vitamin D Binding Protein Genotype**			
1f1f	*Reference*		
1f1s or 1f2	1.65 ± 1.21|0.18		
1s1s, 1s2, or 2,2	0.63 ± 1.09|0.57		

## Data Availability

Data available upon request.

## Supplementary Materials

The following supporting information can be downloaded at https://www.mdpi.com/article/10.3390/nu15194250/s1, Figure S1: Maternal baseline 25(OH)D (first prenatal visit) correlates with offspring birth 25(OH)D. Figure S2: Maternal 25(OH)D 1 month before delivery correlated with offspring birth 25(OH)D. Table S1: 25(OH)D by VDBP genotype reported as median (IQR)
