# Supplementary material for: Vitamin D and Child Neurodevelopment—A Post Hoc Analysis
Source: Nutrients. 2023 Oct 3;15(19):4250. doi: 10.3390/nu15194250 (PMC10574578; doi:10.3390/nu15194250)
Supplement: Supplementary file 1 [file nutrients-15-04250-s001.zip › nutrients-2587278-supplementary.pdf]

Supplemental Figures:

Figure S1: Maternal baseline 25(OH)D (first prenatal visit) correlates with offspring birth 25(OH)D

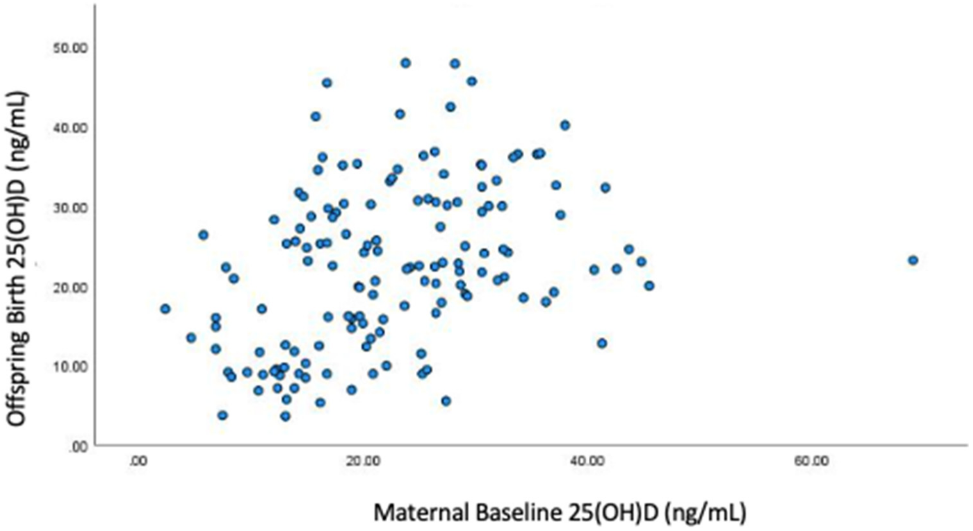

Figure S2: Maternal 25(OH)D 1 month before delivery correlated with offspring birth 25(OH)D

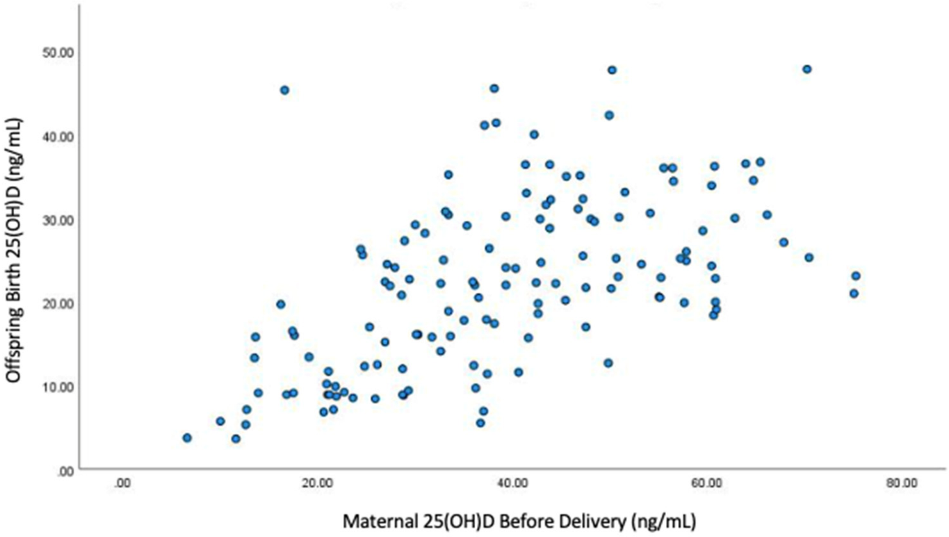

Table S1: 25(OH)D by VDBP genotype reported as median (IQR)

	**1f1f**	**1f1s or 1f2**	**1s1s, 1s2, or 2,2**	**p-value**
**Birth 25(OH)D (ng/mL)**	18.60 (20.50)	24.10 (12.90)	22.30 (13.50)	0.114
**3-5 Year 25(OH)D**	23.00 (12)	27.00 (11)	30.00 (13.00)	0.003
